# SARS-CoV-2 Breakthrough Infection Reveals Platform-Associated Long-Term Immune Remodeling in a Central Asian Cohort

**DOI:** 10.3390/microorganisms14102252

**Published:** 2026-10-04

**Authors:** Lyudmila Akhmaltdinova, Makhabbat Bekbossynova, Kuanysh Dossybayeva, Ainur Tauekelova, Aliya Sailybayeva, Irina Mekhantseva, Iskander Isgandarov, Dimitri Poddighe

**Affiliations:** 1 Department of Biological and Biomedical Sciences, School of Medicine, Nazarbayev University, Astana Z05P3Y4, Kazakhstan; 2 Institute of Life Sciences, Karaganda Medical University, Karaganda M01K6T3, Kazakhstan; 3 Heart Center, Corporate Fund “University Medical Center”, Astana Z05G9F9, Kazakhstan; 4 Breeding and Biotechnology Laboratory, Institute of Plant Biology and Biotechnology, Almaty A15G7X2, Kazakhstan; 5 Department of General Immunology, Asfendiyarov Kazakh National Medical University, Almaty A05H2A6, Kazakhstan; 6 College of Health Sciences, VinUniversity, Hanoi 100000, Vietnam; dimitri.p@vinuni.edu.vn

**Keywords:** COVID-19, breakthrough infection, vaccine platforms, immune memory, CD8^+^ T cells

## Abstract

This study aimed to evaluate platform-specific immune memory trajectories in patients with breakthrough COVID-19 over a 12-month period. This prospective longitudinal study enrolled adults hospitalized with PCR-confirmed COVID-19. Participants were stratified according to prior COVID-19 vaccination status into three groups: unvaccinated, adenoviral-vector-vaccinated, and inactivated-virus-vaccinated. Comprehensive immune profiling included quantification of humoral responses and flow cytometric analysis of T- and B-cell memory subsets. Group differences were assessed using Kruskal–Wallis tests; longitudinal naïve CD8^+^ T-cell dynamics were modeled using linear mixed-effects models; multivariate immune signatures were identified using random forest classification with SHAP analysis. The most distinct cellular finding was a sustained reduction in naïve CD8^+^ T cells in the adenoviral-vector-vaccine group, which persisted for 12 months in patients without pneumonia. Machine learning models demonstrated high discriminative performance for patients without pneumonia (AUC = 0.89–0.92), identifying a specific triad of markers in the adenoviral-vector-vaccine group: decreased naïve CD8^+^ T cells, decreased double-negative effector memory T cells, and increased naïve CD19^+^ B cells. Linear mixed-effects modeling confirmed that contraction of the naïve CD8^+^ T-cell compartment over time was significant exclusively in AdV recipients, whereas pneumonia was the dominant predictor in unvaccinated individuals. No significant temporal or disease-related dynamics were observed in the inactivated vaccine group, whose immune profiles largely overlapped with those of unvaccinated individuals. COVID-19 vaccine platforms induce distinct, durable immune imprints that modulate responses to subsequent breakthrough infections. The adenoviral-vector platform was associated with unique phenotypic remodeling of the T-cell compartment, specifically persistent contraction of the naïve CD8^+^ pool. These findings suggest that the vaccine platform itself, beyond the target antigen, shapes the trajectory of immune competence and long-term memory.

## 1. Introduction

The Coronavirus Disease 2019 (COVID-19) pandemic has been one of the major global health crises of the twenty-first century. Although the emergency phase has ended, several aspects of antiviral immunity to SARS-CoV-2 remain incompletely understood, despite the rapid development of specific vaccines, some of which are based on novel technologies [1,2,3]. However, longitudinal immunological evidence has remained disproportionately focused on mRNA vaccines and high-income country populations. Adenoviral-vector and inactivated vaccines constitute a substantial part of vaccination programs in many low- and middle-income settings, including Central Asia, but their long-term immunological effects following subsequent SARS-CoV-2 infection remain insufficiently characterized [4,5,6].

Kazakhstan’s national vaccination program included the heterologous rAd26/rAd5-based Sputnik V vaccine and several whole-virion inactivated vaccines, including BBIBP-CorV, CoronaVac, and QazVac, making it a suitable setting to address this gap. These platforms differ in antigen composition and innate immune stimulation, which may shape the host response to subsequent viral exposure. Direct longitudinal comparisons after breakthrough infection, however, remain scarce.

Breakthrough infections (i.e., SARS-CoV-2 infections occurring despite prior vaccination) have revealed critical heterogeneity in post-vaccination immune trajectories [7]. Understanding this heterogeneity requires attention to cellular immunity, particularly T-cell memory, a key component of long-term antiviral protection. Whether repeated antigenic exposure, such as breakthrough infection and repeated vaccination, results in “boosted immunity” [8] or, conversely, “functional exhaustion” [9] remains debated; this question is central to understanding long-term immune persistence after breakthrough infection [10].

Most studies of memory T cells rely on cross-sectional cohort designs with short follow-up periods after vaccination or infection (typically 15–90 days), which limit conclusions regarding long-term immune protection [11,12,13]. The present study assessed cellular and humoral immune responses at 1, 6, and 12 months after SARS-CoV-2 infection and compared their dynamics according to prior vaccine platform.

## 2. Materials and Methods

### 2.1. Study Design and Patients

This observational, prospective, and longitudinal study included adult patients (aged ≥18 years) admitted to the hospital because of a diagnosis of acute COVID-19 during the COVID-19 pandemic (study period: between 14 February and 15 June 2022), who had a PCR-confirmed diagnosis of SARS-CoV-2 infection. All the study participants were admitted to the Department of Infectious Diseases (Sub-intensive and Intensive Care Units) of the City Infectious Diseases Center in Astana (Kazakhstan). All the patients included in this study have attended the scheduled follow-up visits until 12 months after their hospital admission related to the acute respiratory disease (T0). In detail, all the study participants received a follow-up assessment at the following time points after the initial hospital admission date (T0): one month (T1), six months (T2), and twelve months (T3).

The following exclusion criteria were applied: lack of informed consent to participate in this study; anamnestic and/or clinical evidence of pre-existing interstitial lung disease; any of the following specific and chronic comorbidities: oncological disorders, hematological disorders, HIV infection, chronic renal failure with dialysis, and any disease previously treated with biological or immunosuppressive therapy; previous treatment with intravenous immunoglobulin or plasma derivatives, and pregnancy.

### 2.2. Clinical, Laboratory and Radiological Data

Demographic characteristics (age and sex) and clinical data, including comorbidities, disease duration, disease onset, body mass index, and symptoms, were collected during routine clinical assessment. At hospital admission, all the patients underwent a complete clinical examination and first-level blood work-up (including the complete blood count, main inflammatory parameters and biochemistry). COVID-19 was diagnosed according to the World Health Organization’s COVID-19 case definitions and, accordingly, all these patients tested positive for SARS-CoV-2 by RT-PCR. All patients with respiratory disease received chest computed tomography (CT) to assess the presence, type, and extent of lung involvement. Based on these radiological findings, patients were classified into one of the following subgroups: COVID-19 with pneumonia (PN) and COVID-19 without pneumonia (NonPN). Therefore, at hospital admission, all these patients with respiratory disease were classified in terms of COVID-19 severity according to the WHO criteria [14].

### 2.3. Vaccination Status

Vaccination status was determined through review of medical records and official vaccination documentation valid at the time of enrollment in this study. The median interval between vaccination and breakthrough infection is shown in Table 1.

Based on this information, the study participants were classified into three groups. Individuals in the unvaccinated group (UnV) had no documented SARS-CoV-2 vaccination. Patients previously immunized with adenovirus-based vaccines (AdVs), specifically with the Sputnik V vaccine, received the heterologous COVID-19 vaccination scheme comprising two immunogenic components: recombinant adenovirus type 26 (rAd26) vector administered as the priming dose and recombinant adenovirus type 5 (rAd5) vector administered as the booster dose. Both vectors were developed by the N. F. Gamaleya Federal Research Center for Epidemiology and Microbiology (Russia) and carried the gene encoding the full-length SARS-CoV-2 spike glycoproteins (rAd26-S and rAd5-S, respectively). Participants in the inactivated vaccine (InV) group received one of the following whole-virion, β-propiolactone–inactivated SARS-CoV-2 vaccines: BBIBP-CorV (marketed as Hayat-Vax or Vero Cell; Sinopharm, Beijing, China, *n* = 24), CoronaVac (Sinovac Biotech, Beijing, China, *n* = 4) or QazVac (Research Institute for Biological Safety Problems, Gvardeyskiy, Kazakhstan, *n* = 7). The inactivated vaccine group was analyzed as a composite category due to comparable immunological mechanisms of action across these formulations.

All the aforementioned vaccines were officially approved and authorized for vaccination in the Republic of Kazakhstan [15].

### 2.4. Serological Assays

As reference markers of immune response development, levels of anti-SARS-CoV-2 IgM, total anti-SARS-CoV-2 IgG, and IgG specific to the receptor-binding domain (RBD) were assessed; the latter is commonly considered a surrogate marker of neutralizing antibodies [16].

All study participants received a specific SARS-CoV-2 serological assessment (by the paramagnetic particle chemiluminescent immunoassay, CLIA) in terms of anti-SARS-CoV-2 IgM (iFlash-SARS-CoV-2 IgM, Shenzhen YHLO Biotech Co. Ltd. (Shenzhen, China), cut-off of 10 AU/mL), anti-SARS-CoV-2 IgG (iFlash-SARS-CoV-2 IgG-S, Shenzhen YHLO Biotech Co. Ltd., cut-off of 10 AU/mL, measuring range of 2.00–3500 AU/mL), and anti-S-RBD SARS-CoV-2 Neutralization Antibody (NAb) (iFlash-2019-nCoV NAb, Shenzhen YHLO Biotech Co. Ltd., cut-off of 10 AU/mL, measuring range of 4.00–800 AU/mL).

### 2.5. Flow Cytometry Analysis

All study participants underwent flow cytometric analysis of peripheral blood mononuclear cells to assess the general lymphocyte populations and, in detail, the T- and B-cell memory compartments (DxFlex cytometer by Beckman Coulter, Brea, CA, USA).

The analysis of lymphocyte subpopulations by flow cytometry was performed on the same day when the blood samples were collected. Peripheral blood mononuclear cells (PBMCs) were isolated from EDTA whole blood (3–4 mL) by using standard protocols routinely applied in immunophenotyping. We analyzed several T-cell subsets: helper T cells (CD3^+^/CD4^+^/CD8^−^), cytotoxic T cells (CD3^+^/CD8^+^/CD4^−^), and double-negative T cells (DNTs, CD3^+^/CD4^−^/CD8^−^). Within these main groups, we also assessed the memory profiles with the following subclassification: naïve T cells (CCR7^+^/CD45RO^−^), effector T cells (Teffs, CCR7^−^/CD45RO^−^), central memory T cells (TCMs, CCR7^+^/CD45RO^+^), and effector memory T cells (TEMs, CCR7^−^/CD45RO^+^). Regarding B cells (CD3^−^/CD19^+^), we analyzed naïve B cells (CD27^−^/IgD^+^), unswitched memory B cells (UnSws, CD27^+^/IgD^+^), switched memory B cells (Sws, CD27^+^/IgD^−^), and “double-negative” B cells (DNBs, CD^27−^/IgD^−^).

### 2.6. Statistical Analysis

Data collection and descriptive analysis were carried out by Microsoft^®^ Excel 2010 for Windows. Intergroup differences in categorical variables were statistically analyzed through chi-square test. Continuous variables were expressed as median with quartiles (Me [Q1; Q3]). Only antibody levels were presented as geometric means with geometric standard deviation factors, calculated using natural logarithm-transformed data. Differences between groups were analyzed by Kruskal–Wallis test with post hoc pairwise comparisons (Dunn’s test). A *p*-value < 0.05 was considered statistically significant. The tables present the overall *p*-values of the Kruskal–Wallis test; the same results are indicated by a dotted line in the figures. *p*-values from post hoc analyses are highlighted in bold in the tables with specification of the groups showing significant differences and are also reported in the text.

To account for the repeated-measures structure of the longitudinal data, linear mixed-effects (LME) models were used to analyze changes in naïve CD8^+^ T-cell counts (CD8^+^CCR7^+^CD45RO^−^ cells) across four time points (T0, T1, T2, and T3). Time point was included as a fixed repeated factor, while vaccine group, age, pneumonia status, and disease severity were evaluated as fixed effects or covariates where appropriate. Subject identification number was included as a random effect to account for within-subject correlations across repeated measurements. Restricted maximum likelihood (REML) estimation with Satterthwaite approximation for degrees of freedom was applied. An autoregressive covariance structure of the first order [AR (1)] was selected for repeated measures based on model fit and longitudinal data characteristics. Interaction terms between time point and selected covariates were additionally explored to assess whether temporal trajectories differed according to age or clinical characteristics.

The statistical analysis and elaboration were processed by Statistica 7.0 (StatSoft, Tulsa, OK, USA), Prism 10 (version 10.6.1, GraphPad Software, Boston, MA, USA), and IBM^®^ SPSS^®^ Statistics (version 25, IBM Corporation, Armonk, NY, USA).

### 2.7. Machine Learning Models

A supervised random forest classifier was applied as an exploratory tool to identify immunological profiles associated with vaccination platform and pneumonia status. Machine-learning analyses were performed using Python (version 3.12.2), scikit-learn (version 1.9), and SHAP (version 0.46). Feature importance was assessed using Gini importance and SHAP (SHapley Additive exPlanations) values [17]. Missing values (2.80% of cell values across the 16 memory-subset markers; concentrated in the B-cell compartment) were imputed using k-nearest neighbors (kNN, k = 5, scikit-learn KNNImputer) fitted separately within each training fold of the cross-validation to avoid data leakage. All immune memory subpopulation markers were included as predictors; vaccination status and pneumonia diagnosis served as target labels, with a composite label created to capture their interaction.

Hyperparameters were optimized by grid search with five-fold cross-validation. The dataset was split into training (80%) and testing (20%) subsets using stratified sampling. Two models were developed: a three-class classifier for vaccination status and a multiclass classifier for combined vaccination–pneumonia subgroups. Model performance was evaluated using precision, recall, F1-score, accuracy, and ROC–AUC.

## 3. Results

### 3.1. Demographic and Baseline Clinical Characteristics of Patients

Based on vaccination status, three groups of study participants were defined, as explained in the previous section. The demographic and clinical characteristics are reported in Table 1.

The incidence of pneumonia was significantly higher in the unvaccinated group compared to all vaccinated groups, while the overall inflammatory status did not differ on average. Participants in the inactivated vaccine group were younger.

Because vaccination groups differed in pneumonia frequency and age distribution, subsequent analyses were stratified by pneumonia status and complemented by mixed-effects modeling to account for repeated measurements and covariates (Table 2A,B).

In the non-pneumonia group, no clear effect of vaccination on disease severity was observed due to the low symptom burden and minimal clinical shifts.

Among patients with pneumonia, lung involvement was reduced in the adenovirus-vaccinated group compared to unvaccinated individuals. Other markers of severity did not show significant differences, despite shorter duration and lower total symptom severity scores in the adenovirus group.

### 3.2. Longitudinal Analysis of Memory T-Cell Populations by Vaccination Status

#### 3.2.1. Non-Pneumonia Patients (Three-Group Comparison)

Basic statistical analyses of memory cell subpopulations across time points are presented in Table 3A,B, while the longitudinal dynamics of individual memory subpopulations are illustrated in Supplementary Figures S1–S5.

In the mild disease group, the acute phase was characterized by shifts in B-cell memory compartments in the unvaccinated group, with significant differences compared with the inactivated vaccine group (UnV vs. InV: CD19 DN, *p* = 0.029; CD19 switched, *p* = 0.008; CD19 unswitched, *p* = 0.022). The AdV group showed an intermediate pattern.

Among all analyzed T- and B-cell memory compartments, naïve CD8^+^ T cells showed the most consistent and durable between-group difference, first becoming evident at 1 month after breakthrough infection and remaining detectable at 6 and 12 months of follow-up (*p* < 0.035).

#### 3.2.2. Pneumonia Patients (Three-Group Comparison)

In acute pneumonia, between-group changes in memory cell subpopulations across time points were less pronounced. Differences were observed in the DNT naïve compartment; however, pairwise comparisons between individual groups did not reach statistical significance (in Table 3B, in Supplementary Figures S1–S5.)

Among the analyzed memory-cell subsets, TEM CD8^+^ T cells showed the most consistent difference between vaccination groups (AdV vs. UnV, 1 and 6 months). Other subsets, including DNT Teff, DNT TEM, CD8 Teff, CD8 naïve, and CD4 naïve cells, differed only at isolated time points. These changes did not show a consistent longitudinal direction and were therefore interpreted as secondary observations.

### 3.3. Linear Mixed-Effects Model: Naïve CD8 T-Cell Dynamics

Because naïve CD8^+^ T cells emerged as the most consistent longitudinal signal in the conventional group comparisons, we further examined their dynamics using linear mixed-effects modeling. This approach was applied to account for repeated measurements within the same individuals and to assess whether changes in the naïve CD8^+^ compartment were independently associated with time point, age, pneumonia status, or their interaction (Table 4).

Linear mixed-effects modeling identified significant effects of time point (*p* = 0.013) and a time point × age interaction (*p* = 0.017) on naïve CD8^+^ T-cell levels exclusively in AdV recipients, while age was a significant predictor across all groups (*p* < 0.001 in each). In the inactivated vaccine group, neither time point nor pneumonia status reached significance. In unvaccinated individuals, pneumonia—rather than time—was the dominant predictor (*p* = 0.004), suggesting that naïve CD8^+^ T-cell dynamics in this group reflect inflammatory burden rather than longitudinal remodeling.

These findings indicate that temporal contraction of the naïve CD8^+^ T-cell compartment is specific to adenoviral-vector vaccination and is not attributable to age alone.

### 3.4. Multivariate Pattern Analysis Using Machine Learning

**Figure 1:**
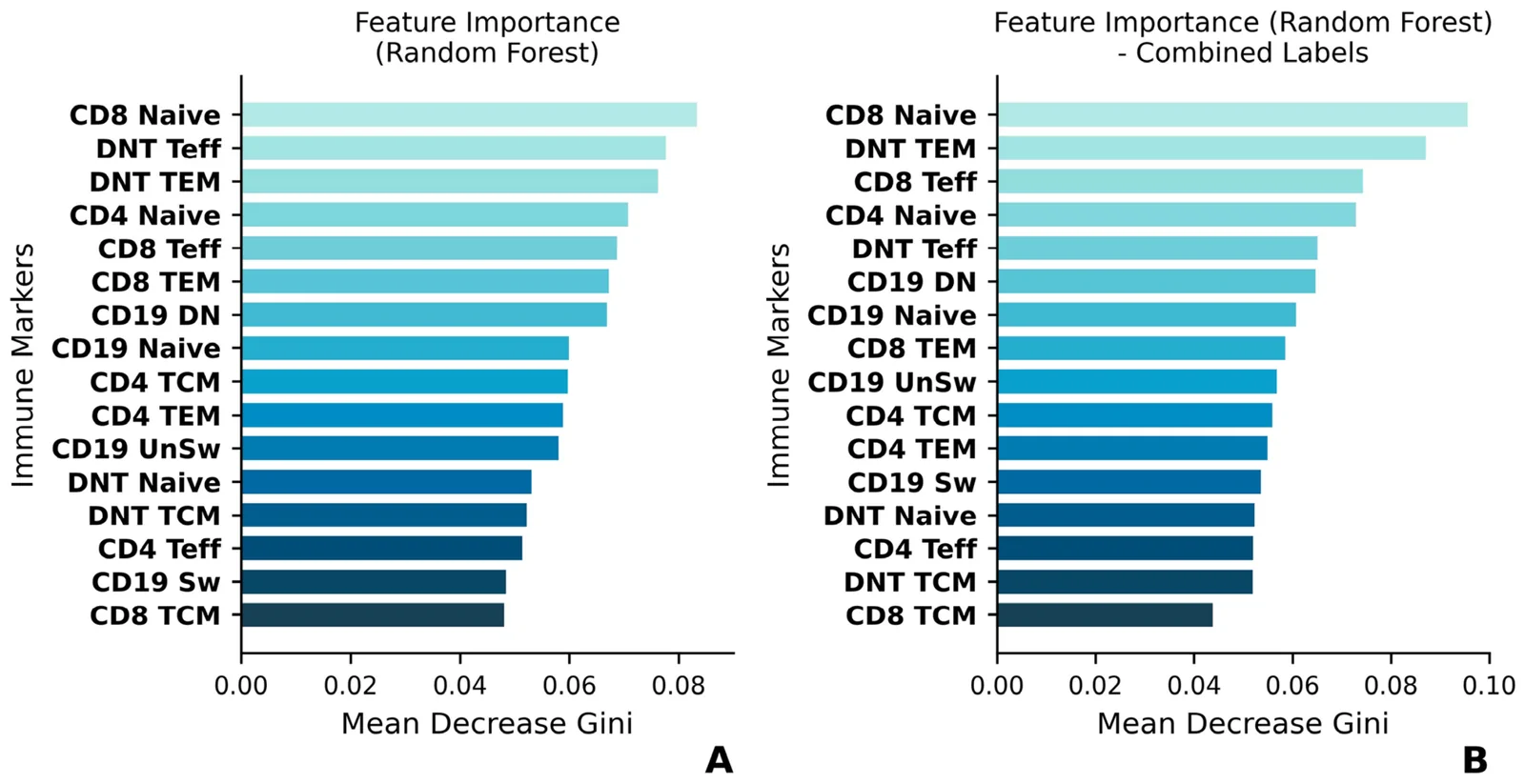
Variable importance based on mean decrease in Gini impurity for (**A**) the vaccination-group classifier and (**B**) the combined vaccination–pneumonia multiclass classifier.

The univariate analyses presented above identified isolated between-group differences in immune cell populations. However, the high dimensionality of the dataset—multiple immune markers measured at four time points across small groups—limits the interpretability of individual comparisons. A random forest classifier was therefore applied to assess whether these markers collectively form stable discriminative patterns across vaccination backgrounds and disease severity. After hyperparameter tuning, two random forest models were evaluated: one assessing the effect of vaccine type, and a multiclass model jointly assessing vaccine type and disease severity. Feature importance was extracted from both models (Figure 1).

The feature importance analysis identified the most influential immunological markers contributing to the classification of vaccination groups and combined vaccination–pneumonia statuses. In both the vaccination-only and combined models, CD8^+^ naïve consistently emerged as the most critical predictor across the decision trees. In the vaccination-only model (Figure 1A), other key contributors included DNT Teff and DNT TEM, which collectively provided significant discriminatory power. When analyzing the combined labels of vaccination and pneumonia status (Figure 1B), the same immunological markers remained influential, with slight shifts in their relative importance. Specifically, DN TEM and CD8 Teff remained the top predictors that consider both vaccination status and pneumonia presence.

**Figure 2:**
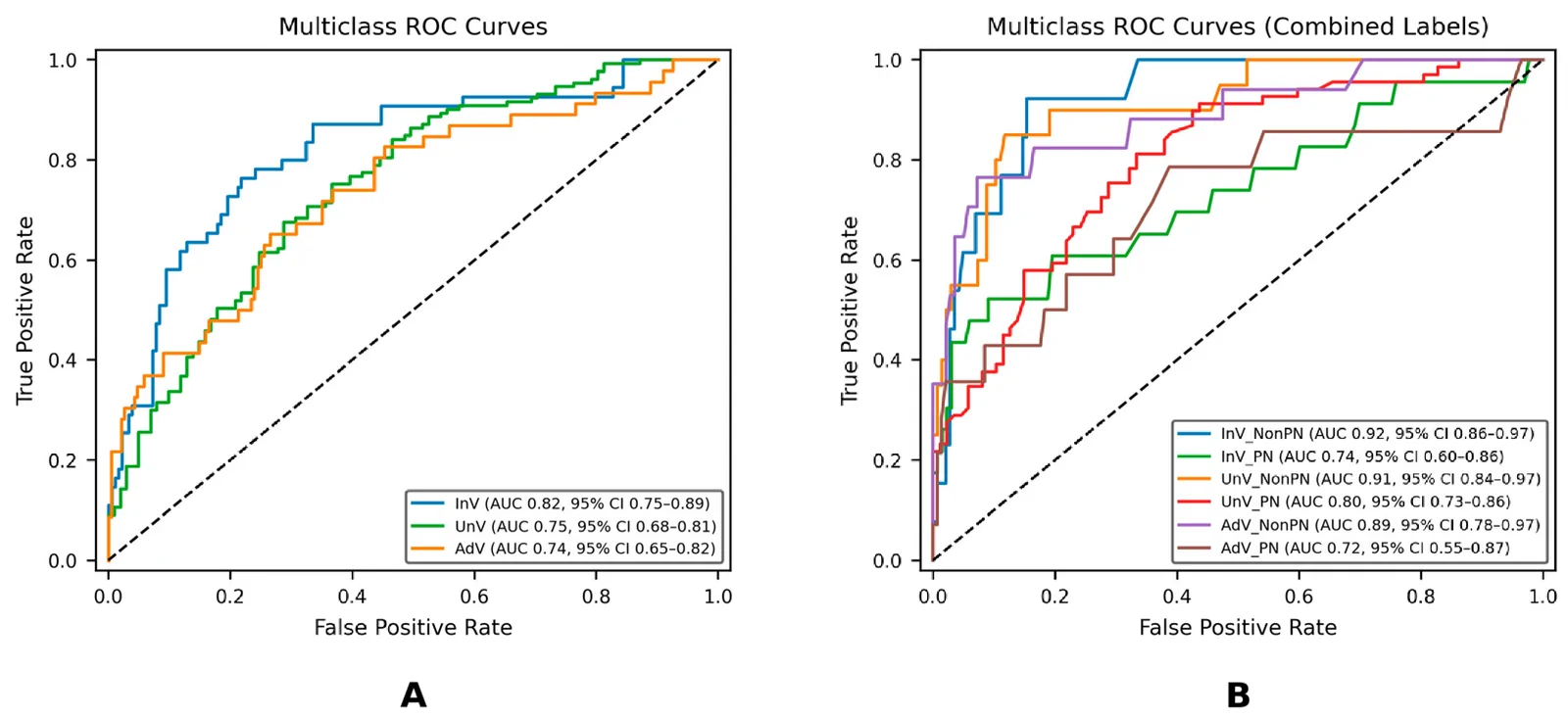
ROC curves for (**A**) the vaccination-group classifier and (**B**) the combined vaccination–pneumonia multiclass classifier. The dotted diagonal lines represent the no-discrimination reference (AUC = 0.5).

Model performance was evaluated using precision, recall, F1-score, overall accuracy, and ROC–AUC analysis (Table 5, Figure 2). Precision, recall, and accuracy describe classification at a fixed decision threshold; ROC–AUC summarizes the model’s ability to separate groups across all possible thresholds and is therefore a more robust indicator of discriminative performance in imbalanced datasets.

In the vaccination-only model, overall accuracy was 68%. The unvaccinated group was identified with high reliability (recall 0.98, precision 0.65). Recall for the AdV and InV groups was considerably lower (0.24 and 0.33, respectively). This asymmetry may reflect a degree of immunological overlap between vaccinated groups, suggesting that recipients of different vaccine platforms shared somewhat more similar immune profiles with each other than with unvaccinated individuals.

Incorporating pneumonia status alongside vaccination group substantially improved discriminative performance (Figure 2). The highest discrimination was achieved for uncomplicated cases: InV NonPN (AUC = 0.92, 95% CI: 0.86–0.97), UnV NonPN (AUC = 0.91, 95% CI: 0.84–0.97), and AdV NonPN (AUC = 0.89, 95% CI: 0.78–0.97). Among complicated cases, performance was moderate: UnV PN (AUC = 0.80, 95% CI: 0.73–0.86), InV PN (AUC = 0.74, 95% CI: 0.60–0.86), and AdV PN (AUC = 0.72, 95% CI: 0.55–0.87). The weaker separation of vaccinated individuals with pneumonia suggests that severe disease attenuates platform-specific immune signatures, driving immunological convergence across groups—a pattern consistent with the univariate findings described above.

**Figure 3:**
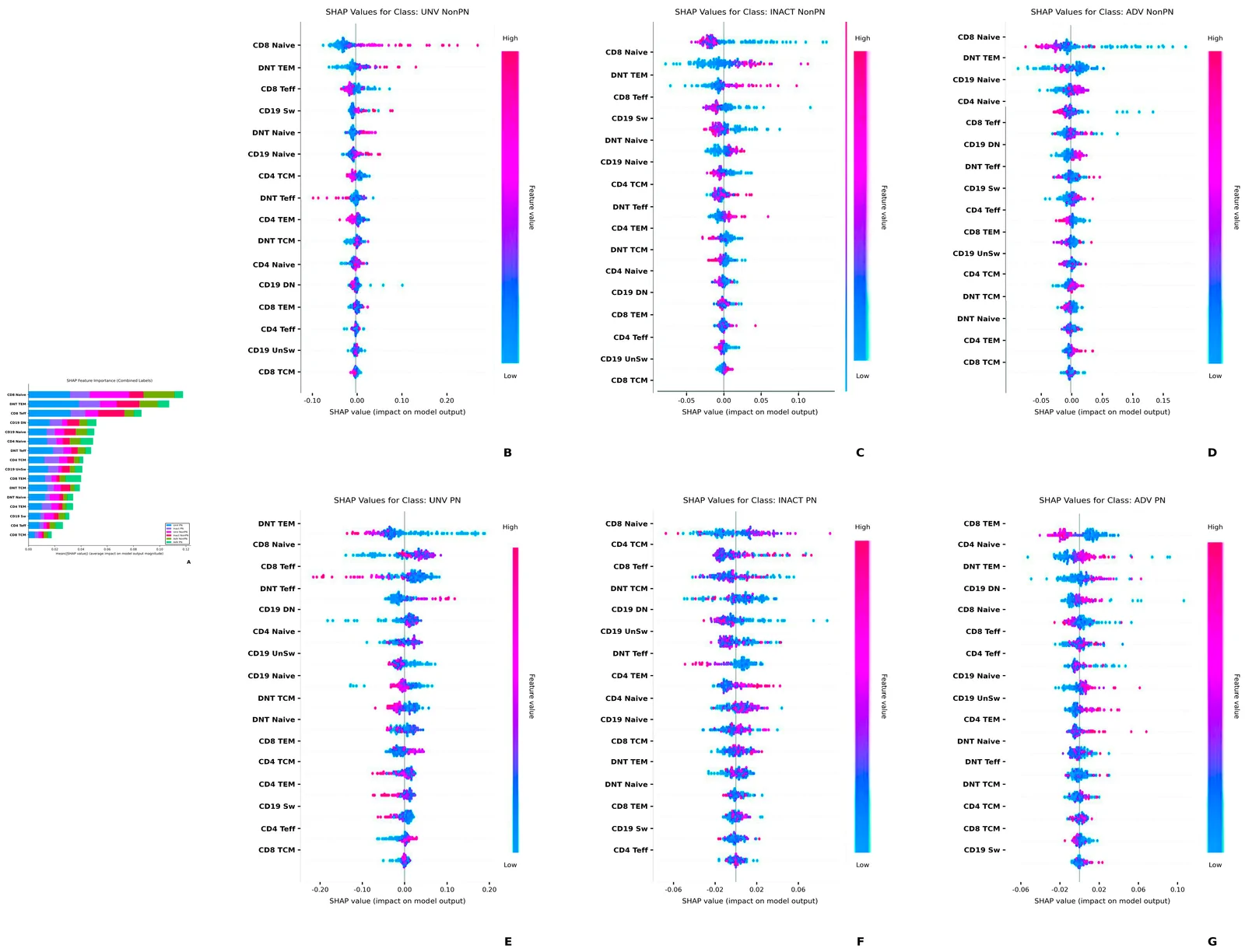
SHAP feature importance for the multiclass model: (**A**) overall feature importance and (**B**–**G**) class-specific SHAP values.

To identify the markers driving these patterns, SHAP analysis was applied, quantifying each variable’s contribution to individual- and group-level predictions (Figure 3). In the beeswarm plots, the horizontal axis reflects the magnitude of each variable’s contribution; point color indicates the observed value of that variable (blue: low, red: high). SHAP analysis confirmed CD8^+^ naïve T cells as the dominant discriminatory feature and revealed group-specific immune signatures.

In uncomplicated cases, the specific immune signature was observed in the AdV group, characterized by decreased naïve CD8^+^ T cells, decreased DNT TEM, and increased naïve CD19^+^ B cells. By contrast, the UnV and InV groups showed a largely opposite and mutually similar pattern, with higher naïve CD8^+^ T cells, higher DNT TEM, and lower CD8 Teff.

In pneumonia cases, immune patterns were more heterogeneous, with no consistent directional pattern identified. We extended the model using SHAP, which enables assessment of both global and local contributions of each variable. The results are presented as a ranked importance plot visualized as a beeswarm scatter plot. In this representation, the horizontal axis indicates the SHAP value, while point color reflects the magnitude of each variable (blue for low values and red for high values). SHAP analysis identified the variables with the strongest influence (Figure 3A) and visualized memory patterns characteristic of each scenario (Figure 3B–G).

## 4. Discussion

Breakthrough COVID-19 provides an opportunity to examine the immune response generated by different vaccine platforms during subsequent SARS-CoV-2 exposure, potentially revealing differences in post-infection immunity that are not captured by conventional measures of vaccine effectiveness [18,19]. The vaccines used for mass immunization in Kazakhstan were based on platforms developed in China, Russia, and Kazakhstan, all of which are represented in the present study. The effects of COVID-19 severity on memory-cell populations were examined separately in our earlier publications and were not the primary focus of the current analysis [20,21].

The study was conducted between 14 February and 15 June 2022, during a shift in the SARS-CoV-2 variant landscape in Kazakhstan. National genomic surveillance data indicate that Omicron cases peaked in February 2022, with the BA.1 and BA.2 sublineages co-circulating from March onward. Both sublineages carry multiple spike mutations relative to the ancestral strain used in vaccine antigen design, which can reduce antibody-mediated neutralization irrespective of the vaccine platform. Our results should therefore be interpreted in the context of substantial antigenic drift and widespread humoral immune escape during the study period [22].

The main finding of the 12-month follow-up was a sustained reduction in naïve CD8^+^ T cells among recipients of the adenoviral-vector vaccine who developed breakthrough COVID-19 without pneumonia. Linear mixed-effects modeling showed that this trajectory could not be attributed to age alone and was specific to the AdV group among patients with uncomplicated disease. Naïve CD8^+^ T-cell levels were also lower in patients with pneumonia than in those without, across all vaccination categories—consistent with our earlier observations [20,21]—and pneumonia was an independent predictor of naïve CD8^+^ T-cell levels in unvaccinated participants (*p* = 0.004), confirming the association between this compartment and COVID-19 severity. Machine-learning analysis, used as a complementary approach, showed that classification was more successful among patients without pneumonia, primarily separating unvaccinated participants from AdV recipients; the AdV group displayed coordinated changes across several memory compartments, with reduced naïve CD8^+^ T cells contributing substantially to group discrimination. Classification was less reliable among patients with pneumonia, particularly for recipients of inactivated vaccines, who showed little separation from unvaccinated participants.

These findings can be placed alongside prior reports on the two vaccine platforms represented in our cohort. Inactivated COVID-19 vaccines, including CoronaVac, have been reported to increase CD4^+^ and CD8^+^ memory T cells as well as memory B-cell populations [23], with seroconversion rates of approximately 50% indicating a comparatively limited humoral response [24,25] and a more rapid decline in antibody levels than other platforms—possibly reflecting transient antigen exposure and limited persistence of germinal-center reactions—alongside a Th1-biased response to multiple viral antigens [26,27,28]. Sputnik V, based on the non-replicating adenoviral vectors Ad26 and Ad5, has shown real-world effectiveness exceeding 97% and induces both humoral and cellular responses [29]; in our cohort, it was likewise associated with higher antibody levels than natural infection alone or inactivated vaccination, in patients with and without pneumonia alike [30].

Several non-mutually exclusive mechanisms may explain the selective naïve CD8^+^ contraction observed in the AdV group. First, vaccine platforms differ in the degree to which they engage the CD8^+^ compartment: inactivated whole-virion vaccines predominantly induce CD4^+^ responses, with CD8^+^ responses detected less frequently, whereas mRNA and adenoviral-vector vaccines encode the spike antigen and engage both CD4^+^ and CD8^+^ T cells [31]. This greater baseline CD8^+^ engagement may itself predispose the AdV group to more pronounced antigen-driven recruitment and differentiation of naïve CD8^+^ T cells, with persistent or repeated antigenic stimulation contributing to functional changes in this compartment [32,33]. Second, redistribution of CD8^+^ T cells from peripheral blood to lymphoid organs or affected tissue offers a complementary explanation [34]. SARS-CoV-2-specific CD8^+^ T cells have been detected at higher frequencies in the upper respiratory tract and lung than in peripheral blood, remaining functional for up to 12 months after antigen exposure even when circulating populations are barely measurable [31]. Such redistribution could plausibly contribute to the lower circulating levels seen in patients with pneumonia, though its relevance to mild breakthrough infection is less clear and warrants further study. Third, vaccination may alter not only the size but the functional quality of the T-cell response to subsequent infection: in a comparative study of vaccinated and unvaccinated individuals recovering from mild COVID-19, unvaccinated patients showed a more effector-oriented baseline CD8^+^ profile (higher CD69 and IFN-γ), while vaccinated patients with breakthrough infection showed higher perforin, TNF-α, and IL-10 expression in CD8^+^ T cells after spike restimulation, together with greater CD137^+^/CD69^+^ CD4^+^ T-cell activation [35]. Our results suggest that this qualitative reshaping of the post-infection T-cell response may itself differ between vaccine platforms. This remains hypothetical, however, as antigen-specific CD8^+^ T-cell responses and functional activity were not directly measured in this study.

More broadly, our findings add to a growing body of evidence that vaccine platforms differ not only in antigen delivery but in their wider effects on immune regulation. Platform-dependent tolerogenic and regulatory immune states have previously been described primarily in the context of mRNA and inactivated vaccines [36]; our data suggest that a comparable platform-specific imprint extends to adenoviral-vector vaccines as well, manifesting here as a selective and sustained contraction of the naïve CD8^+^ compartment. While the available evidence is insufficient to establish a direct mechanistic link between these broader regulatory effects and the reduction observed in our cohort, the consistency of platform-specific immune signatures across studies and vaccine types strengthens the case that vaccine platform itself—beyond its role in eliciting protective immunity—leaves a distinct and lasting imprint on immune-cell composition. To our knowledge, this is the first longitudinal analysis focused on immune-memory populations after breakthrough COVID-19 in recipients of an adenoviral-vector vaccine.

## 5. Conclusions

Breakthrough COVID-19 enables qualitative assessment of vaccine-induced immune modulation beyond conventional effectiveness metrics. Adenoviral-vector vaccination was associated with persistently higher antibody levels during long-term follow-up and demonstrated indirect signals of inactivated vaccine group performance compared with other platforms. Overall, the clearest platform-associated pattern was observed in adenoviral-vector-vaccine recipients. Inactivated-vaccine recipients showed limited differences from unvaccinated individuals, whereas the AdV group displayed a more distinctive immune profile, centered on reduced naïve CD8^+^ T-cell levels. This feature was evident in uncomplicated breakthrough infection and remained relevant in pneumonia cases, suggesting that the naïve CD8^+^ compartment may represent a sensitive marker of both disease-related and vaccine-platform-associated immune remodeling. Compared with patients without pneumonia, those with pneumonia showed broader and less platform-specific immune changes, suggesting that clinically manifest lung involvement partially obscures vaccine-associated memory signatures. In this setting, immune profiles became more convergent across vaccination groups.

## 6. Limitations

Several limitations should be acknowledged. First, the sample size was limited, particularly within the vaccination subgroups and non-pneumonia strata. This was partly related to the strict inclusion criteria, including PCR-confirmed COVID-19 and completion of the full 12-month follow-up. Second, the adenoviral-vector group consisted exclusively of Sputnik V recipients. Third, the inactivated vaccine group was analyzed as a composite category, which may have masked differences between individual inactivated vaccine products. Fourth, pre-vaccination immune profiles were not available. As a result, we could not directly compare immune-cell distributions before vaccination and after breakthrough infection. Finally, this was an observational single-center study, and causal inference was not possible. The associations between vaccine platform and immune phenotype should therefore be considered hypothesis-generating and require confirmation in larger longitudinal cohorts with pre-vaccination sampling, antigen-specific T-cell assays, and broader representation of vaccine platforms. Immunophenotyping panel (16 markers × four time points × pneumonia strata) was not corrected for multiple comparisons and should be interpreted as exploratory; the primary naïve CD8^+^ T-cell finding was independently confirmed by a pre-specified linear mixed-effects model and remained significant under FDR correction within that comparison family.

## Figures and Tables

**Table 1:** Demographic and baseline clinical characteristics of patients stratified by vaccine type.

Parameter	UnV	AdV	InV	p [AU; AI; UI]
Number of patients (*n*)	112	38	35	-
First dose-to-infection interval (months)	-	10.0 [8.0; 10.0]	8.0 [6.75; 9.0]	>0.05
Pneumonia (%)	82.1	60.5	68.6	**0.018** [**<0.001**; >0.05; **0.004**]
Age (years)	62.64 [52.98; 68.06]	63.79 [54.99; 67.68]	58.37 [36.95; 64.26]	**0.02** [>0.05; >0.05; **0.02**]
Gender M/F (%)	36.6/63.4	39.5/60.5	42.9/57.1	>0.05
BMI (kg/m^2^)	29.7 [25.9; 33.1]	27.34 [25.2; 31.2]	29.1 [25.4; 30.4]	>0.05
Duration (d)	8.0 [6.0; 12.0]	6.5 [1.0; 8.0]	7.0 [5.0; 13.0]	**0.02** [**0.01**; >0.05; >0.05]
Deaths *n* (%)	1 (0.9%)	1 (2.6%)	0 (0%)	>0.05
Post-COVID-19 symptoms (%)	75.0	71.1	62.9	>0.05
Total symptom severity score at T0 (points)	5 [4; 6]	4 [3; 5.75]	5 [4; 6]	>0.05
CRP at T0 (mg/L)	1.08 [0.43; 3.51]	1.09 [0.16; 5.17]	1.17 [0.51; 3.82]	>0.05
WBC at T0 (10^9^/L)	6.21 [4.81; 8.88]	6.47 [5.75; 7.7]	5.7 [4.9; 6.62]	>0.05
Ferritin at T0 (ng/mL)	326.9 [129.5; 575.7]	176.0 [79.0; 303.8]	239.17 [116.2; 372.3]	>0.05
T0-SARS-CoV-2 IgM (AU/mL)	2.41 ± 6.91	0.71 ± 4.28	2.69 ± 6.08	**0.01** [**0.01**; **0.01**; >0.05]
T0-SARS-CoV-2 IgG (AU/mL)	38.66 ± 15.88	409.89 ± 5.65	161.29 ± 8.49	**0.01** [**0.01**; >0.05; **0.03**]
T0-SARS-CoV-2 IgG-RBD (AU/mL)	19.53 ± 11.72	314.71 ± 5.31	64.24 ± 13.48	**0.01** [**0.01**; **0.01**; **0.03**]
T1-SARS-CoV-2 IgG (AU/mL)	300.89 ± 5.86	2141.96 ± 2.56	915.47 ± 5.80	**0.01** [**0.01**; >0.05; **0.01**]
T1-SARS-CoV-2 IgG-RBD (AU/mL)	35.18 ± 9.66	748.52 ± 3.34	162.32 ± 10.17	**0.01** [**0.01**; **0.01**; **0.04**]
T2-SARS-CoV-2 IgG (AU/mL)	384.89 ± 4.37	1428.18 ± 2.49	582.00 ± 5.00	**0.01** [0.01; 0.04; >0.05]
T2-SARS-CoV-2 IgG-RBD (AU/mL)	65.37 ± 7.46	569.50 ± 2.67	133.66 ± 11.31	**0.04** [**0.01**; **0.01**; >0.05]
T3-SARS-CoV-2 IgG (AU/mL)	346.30 ± 4.90	1417.46 ± 2.32	388.50 ± 6.09	**0.01** [**0.01**; **0.01**; >0.05]
T3-SARS-CoV-2 IgG-RBD (AU/mL)	56.65 ± 6.82	412.19 ± 3.22	117.92 ± 6.07	**0.01** [**0.01**; >0.05; >0.05]

**Notes**: Data are presented as median [Q1; Q3] or geometric mean ± geometric standard deviation factor, as appropriate; categorical variables are presented as percentages or n (%). Overall p-values are followed, where applicable, by post hoc pairwise p-values in square brackets in the following order: [AU; AI; UI]. Bold values indicate statistically significant differences in pairwise comparisons (*p* < 0.05). The total symptom severity score was constructed based on a clinical interview and a questionnaire comprising 21 symptoms, each assessed using a binary (0–1) scale: weakness, headache, fever, sore throat, dyspnea, feeling of shortness of breath, chest pain, myalgia, joint pain, sleep disorder, anosmia, ageusia, diarrhea, cutaneous manifestations, hair loss, loss of appetite, vertigo, heartbeat, fatigue, decreased memory, and anxiety. T0, baseline; T1, 1 month; T2, 6 months; T3, 12 months. **Abbreviations**: AdV, adenovirus-vectored vaccine; AI, AdV vs. InV; AU, AdV vs. UnV; AU/mL, arbitrary units per milliliter; BMI, body mass index; CRP, C-reactive protein; IgG, immunoglobulin G; IgM, immunoglobulin M; InV, inactivated vaccine; M/F, male/female; PN, pneumonia; RBD, receptor-binding domain; UI, UnV vs. InV; UnV, unvaccinated; WBC, white blood cell count.

**Table 2:** (A) Demographic and clinical characteristics of the non-pneumonia group. (B) Demographic and baseline clinical characteristics of the pneumonia group.

**(A)**				
**Parameter**	**UnV NonPN**	**AdV NonPN**	**InV NonPN**	**p [AU; AI; UI]**
Number of patients *(n)*	20	15	11	-
Age (years)	54.02 [43.44; 62.19]	63.54 [58.09; 68.34]	59.76 [49.9; 65.23]	>0.05
Gender M/F (%)	25/75	33.3/66.7	54.5/45.5	>0.05
BMI (kg/m^2^)	27.02 [25.5; 29.88]	27.1 [24.5; 31.6]	29.4 [25.34; 33.6]	>0.05
Duration (d)	5 [0; 8]	4 [0; 7]	4 [0; 7]	>0.05
Deaths *n* (%)	0 (0%)	0 (0%)	0 (0%)	>0.05
Total symptom severity score at T0 (points)	4 [3; 5]	4 [3; 6]	4 [3; 6]	>0.05
CRP at T0 (mg/L)	0.27 [0.11; 0.69]	0.3 [0.08; 2.75]	0.64 [0.28; 2.6]	>0.05
WBC at T0 (10^9^/L)	7.05 [5.28; 9.43]	6.7 [5.69; 8.15]	5.6 [4.25; 6.27]	>0.05
Ferritin at T0 (ng/mL)	129.5 [89.13; 335.7]	122.9 [64.9; 204.6]	239.1 [155.75; 273.15]	>0.05
T0-SARS-CoV-2 IgM (AU/mL)	1.32 ± 3.17	0.61 ± 2.68	0.77 ± 2.67	>0.05
T0-SARS-CoV-2 IgG (AU/mL)	182.1 ± 15.8	839.13 ± 2.94	258.88 ± 7.82	>0.05
T0-SARS-CoV-2 IgG-RBD (AU/mL)	68.07 ± 13.26	489.59 ± 2.87	162.45 ± 7.5	**0.008** [**0.006**; >0.05; >0.05]
T1-SARS-CoV-2 IgG (AU/mL)	288.61 ± 8.6	2477.64 ± 1.52	1633.9 ± 2.19	**0.005** **[<0.001**; >0.05; >0.05]
T1-SARS-CoV-2 IgG-RBD (AU/mL)	81.75 ± 11.98	1018.59 ± 1.48	410.39 ± 3.04	**0.003** [0.000; >0.05; >0.05]
T2-SARS-CoV-2 IgG (AU/mL)	484.75 ± 2.91	1914.55 ± 1.95	1189.97 ± 2.93	**0.002** [**0.002**; >0.05; >0.05]
T2-SARS-CoV-2 IgG-RBD (AU/mL)	116.31 ± 4.53	740.49 ± 1.42	474.96 ± 2.19	**0.001** [**<0.001**; >0.05; >0.05]
T3-SARS-CoV-2 IgG (AU/mL)	190.21 ± 6.75	1954.48 ± 2	913.29 ± 3.85	**0.0032** [**0.003**; >0.05; >0.05]
T3-SARS-CoV-2 IgG-RBD (AU/mL)	36.1 ± 6.61	572.86 ± 2.27	229.5 ± 5.69	**0.0068** [**0.008**; >0.05; >0.05]
**(B)**				
**Parameter**	**UnV PN**	**AdV PN**	**InV PN**	**p [AU; AI; UI]**
Number of patients *(n)*	92	23	24	-
PN severity category, II/III (%)	80/20	100/0	87/13	>0.05
Lung damage (%)	16.0 [4.0; 34.3]	4 [0.0; 20.0]	7.0 [0.0; 26.0]	**0.01** [**0.01**; >0.05; >0.05]
Age (years)	64.49 [56.81; 69.05]	64.02 [49.54; 67.71]	59.75 [48.3; 65.6]	>0.05
Gender M/F (%)	39.1/60.9	43.5/56.5	37.5/62.5	>0.05
BMI (kg/m^2^)	30.3 [26; 34]	27.58 [25.54; 31.25]	28.82 [24.7; 29.9]	>0.05
Duration (d)	9 [6; 14]	7 [4; 10]	8.5 [6.5; 14]	>0.05
Deaths *n* (%)	1 (1.1%)	1 (4.0%)	0 (0.0%)	>0.05
Total symptom severity score at T0 (points)	5 [4; 6]	4 [3; 6]	5 [4; 6]	>0.05
CRP at T0 (mg/L)	1.50 [0.54; 3.86]	2.71 [0.35; 7.79]	1.43 [0.55; 6.53]	>0.05
WBC at T0 (10^9^/L)	6.15 [4.87; 8.55]	6.33 [5.77; 7.80]	5.8 [5.15; 6.86]	>0.05
Ferritin at T0 (ng/mL)	371.4 [181.3; 640.25]	249.1 [118.1; 335.2]	256.7 [109.1; 380.3]	>0.05
T0-SARS-CoV-2 IgM (AU/mL)	2.76 ± 7.76	0.79 ± 5.63	4.74 ± 6.55	**0.005** [**0.005**; 0.02; >0.05]
T0-SARS-CoV-2 IgG (AU/mL)	27.56 ± 14.35	251.49 ± 6.93	117.83 ± 9.01	**0.006** [**0.002**; >0.05; >0.05]
T0-SARS-CoV-2 IgG-RBD (AU/mL)	15.34 ± 10.56	232.84 ± 7.02	37.8 ± 16.07	**<0.001** [**0.004**; **<0.001**; >0.05]
T1-SARS-CoV-2 IgG (AU/mL)	304.91 ± 5.23	1721.76 ± 4.15	648.69 ± 7.87	**0.005** [**0.007**; >0.05; >0.05]
T1-SARS-CoV-2 IgG-RBD (AU/mL)	26.88 ± 8.62	471.53 ± 6.14	92.81 ± 14.1	**0.005** [**0.004**; >0.05; >0.05]
T2-SARS-CoV-2 IgG (AU/mL)	365.02 ± 4.73	1217.19 ± 2.72	403.44 ± 6.06	**0.005** [**0.004**; >0.05; >0.05]
T2-SARS-CoV-2 IgG-RBD (AU/mL)	57.37 ± 8.11	493.52 ± 3.25	70.12 ± 16.25	**<0.001** [**0.007**; **<0.001**; >0.05]
T3-SARS-CoV-2 IgG (AU/mL)	398.99 ± 4.43	1126.82 ± 2.42	208.79 ± 6.89	**0.016** [>0.05; **0.02**; >0.05]
T3-SARS-CoV-2 IgG-RBD (AU/mL)	62.89 ± 6.88	325.83 ± 3.85	73.39 ± 6.10	**0.019** [**0.015**; >0.05; >0.05]

**Notes:** (A) Participants without pneumonia (NonPN); (B) participants with pneumonia (PN). Data are presented as median [Q1; Q3] or geometric mean ± geometric standard deviation factor, as appropriate; categorical variables are presented as percentages or *n* (%). Overall *p*-values are followed, where applicable, by post hoc pairwise *p*-values in square brackets in the following order: [AU; AI; UI]. Statistically significant *p*-values (*p* < 0.05) are shown in bold. T0, baseline; T1, 1 month; T2, 6 months; T3, 12 months. **Abbreviations:** AdV, adenovirus-vectored vaccine; AU, AdV vs. UnV; AI, AdV vs. InV; BMI, body mass index; CRP, C-reactive protein; IgG, immunoglobulin G; IgM, immunoglobulin M; InV, inactivated vaccine; NonPN, without pneumonia; PN, pneumonia; RBD, receptor-binding domain; UI, UnV vs. InV; UnV, unvaccinated; WBC, white blood cell count; AU/mL, arbitrary units per milliliter.

**Table 3:** (A) T- and B-cell memory compartments in the non-pneumonia group. (B) T- and B-cell memory compartments in the pneumonia group.

**(A)**				
**Parameter** **(10^3^/L)**	**UnV NonPN** **(*n* = 20)**	**AdV NonPN** **(*n* = 15)**	**InV NonPN** **(*n* = 11)**	***p*** **-value**
T0—acute phase				
CD4 naïve	387.6 [196.39; 612.1]	218.1 [86.1; 250.4]	233.8 [180.8; 629.1]	>0.05
CD4 TCM	250.3 [166.16; 510.2]	215.6 [123.6; 315.6]	273.4 [146.5; 413.0]	>0.05
CD4 Teff	19.3 [13.81; 25.4]	17.4 [6.4; 30.0]	13.4 [6.6; 21.4]	>0.05
CD4 TEM	266.1 [179.6; 397.0]	231.2 [129.3; 310.1]	205.4 [130.9; 248.2]	>0.05
CD8 naïve	107.3 [56.47; 249.2]	57.2 [17.7; 118.1]	69.1 [46.6; 106.0]	>0.05
CD8 TCM	31.2 [20.26; 48.0]	25 [15.2; 41.9]	38.9 [23.3; 76.3]	>0.05
CD8 Teff	104.7 [54.13; 196.0]	121.7 [45.8; 251.1]	51.1 [29.3; 68.3]	>0.05
CD8 TEM	177.7 [114.32; 345.1]	168.2 [79.1; 227.0]	148.7 [104.4; 190.5]	>0.05
DNT naïve	26.4 [6.91; 45.4]	18.5 [12.9; 37.0]	28.8 [16.9; 42.2]	>0.05
DNT TCM	3.2 [1.85; 6.1]	3.9 [1.7; 7.1]	6.3 [2.1; 9.2]	>0.05
DNT Teff	14.6 [5.7; 24.0]	11.3 [7.5; 24.3]	14.2 [4.6; 17.0]	>0.05
DNT TEM	6.4 [3.1; 10.3]	3.7 [2.7; 6.4]	2.7 [2.1; 5.1]	>0.05
CD19 DN	7.2 [4.46; 10.4]	5.6 [2.5; 13.1]	3.9 [3.1; 4.6]	**0.026**
CD19 naïve	138.8 [55.16; 296.8]	95.1 [46; 177.1]	47.8 [30.6; 71.1]	>0.05
CD19 Sw	53.4 [39.15; 76.4]	35.8 [22.5; 67.3]	28.6 [22.1; 32.7]	**0.008**
CD19 UnSw	21.96 [14.56; 24.58]	16.9 [9; 29.4]	8.3 [7.5; 14.6]	**0.022**
1 month				
CD4 naïve	292.3 [167.62; 464.1]	276.1 [181.3; 319.4]	254.5 [145.5; 580.0]	>0.05
CD4 TCM	193.3 [130.88; 256.3]	259.3 [153.4; 398.7]	242.1 [227.7; 410.4]	>0.05
CD4 Teff	14.1 [9.22; 40.4]	21.1 [14.5; 53.4]	14.5 [8.7; 26.4]	>0.05
CD4 TEM	218.2 [149.91; 249.2]	221.8 [180.5; 428.3]	241.6 [224.2; 304.2]	>0.05
CD8 naïve	92.6 [40.25; 199.3]	31.3 [17.7; 54.1]	68.4 [30.7; 141.4]	**0.003**
CD8 TCM	29 [14.32; 40.7]	34.7 [12.3; 71.2]	34.2 [21.1; 45.6]	>0.05
CD8 Teff	91.9 [58.4; 162.2]	167.9 [97.2; 255.6]	53.8 [43.1; 94.4]	**0.014**
CD8 TEM	141.9 [118.82; 261.0]	130.7 [79.8; 310.5]	148.1 [83.4; 260.6]	>0.05
DNT naïve	40.3 [10.65; 59.0]	23.4 [15.5; 29.6]	46.3 [25.7; 56.7]	>0.05
DNT TCM	2.8 [0.98; 5.2]	3.3 [1.2; 4.7]	5.9 [2.2; 8.2]	>0.05
DNT Teff	14.3 [6.99; 21.7]	14.6 [10.7; 30.2]	11.41 [6.6; 14.8]	>0.05
DNT TEM	4.6 [2.38; 8.8]	2.3 [1.5; 4.4]	2.2 [2.0; 5.1]	>0.05
CD19 DN	4.6 [3.26; 6.2]	5.7 [4.3; 7.5]	4.1 [3.5; 5.2]	>0.05
CD19 naïve	86.8 [62.71; 133.4]	102.1 [66.2; 148.9]	38.9 [30.6; 110.4]	>0.05
CD19 Sw	27.2 [15.58; 42.5]	30.1 [21.5; 60.4]	31.1 [19.7; 37.4]	>0.05
CD19 UnSw	15 [10.12; 34.1]	17.5 [12.6; 30.1]	13.3 [7.1; 22.2]	>0.05
6 months				
CD4 naïve	346.2 [213.4; 493.3]	242.2 [198.4; 361.6]	351.0 [177.9; 522.6]	>0.05
CD4 TCM	212.4 [179.4; 367.7]	318.8 [266.7; 386.2]	262.2 [167.1; 392.5]	>0.05
CD4 Teff	21.1 [6.6; 55.4]	18.1 [10.3; 31.5]	17.1 [9.5; 47.5]	>0.05
CD4 TEM	198 [146.8; 219.9]	247.9 [203.8; 297.9]	140.0 [60.9; 253.1]	>0.05
CD8 naïve	144.8 [67.3; 207.1]	42 [19.2; 102.5]	95.3 [28.5; 200.9]	**0.035**
CD8 TCM	43.2 [26.1; 84.9]	57.7 [44.6; 73.3]	80.4 [59.7; 118.5]	>0.05
CD8 Teff	120.8 [60; 176.9]	130.3 [90.5; 241.2]	86.7 [70.4; 215.2]	>0.05
CD8 TEM	146.1 [82; 228.4]	141.5 [105.4; 314.5]	163.3 [47.2; 294.1]	>0.05
DNT naïve	23.7 [13.2; 41]	23.4 [17.1; 34.3]	34.2 [24.6; 68.2]	**0.042**
DNT TCM	4.9 [2.7; 9.8]	4.5 [3.6; 5.2]	11.7 [3.5; 23.2]	>0.05
DNT Teff	20.5 [12.4; 29.7]	16.1 [9.4; 22.5]	15.3 [7.6; 24.0]	>0.05
DNT TEM	4.9 [4; 7.2]	5.3 [2.4; 6.3]	4.1 [1.4; 8.6]	>0.05
CD19 DN	4.65 [1.68; 6.99]	4.77 [3.89; 7.82]	4.04 [2.18; 6.01]	>0.05
CD19 naïve	70.21 [46.22; 144.43]	102.51 [63.59; 125.5]	81.94 [49.24; 123.34]	>0.05
CD19 Sw	37.46 [25.5; 46.64]	35.08 [31.71; 47.61]	38.16 [24.81; 49.04]	>0.05
CD19 UnSw	15.63 [12.44; 29.27]	18.61 [15.63; 30.56]	22.4 [11.74; 30.17]	>0.05
		12 months		
CD4 naïve	341.6 [244.1; 607.7]	244.8 [124.9; 388.5]	480.2 [212.5; 653.1]	>0.05
CD4 TCM	239.4 [190.4; 323]	320.1 [221.0; 439.7]	248.4 [235.8; 389.5]	>0.05
CD4 Teff	20.6 [7.2; 27.6]	18.1 [7.2; 44.7]	12.7 [9.2; 25]	>0.05
CD4 TEM	214.3 [142.6; 281.8]	162.5 [104.1; 230.6]	223.1 [123.8; 303.1]	>0.05
CD8 naïve	140.8 [64.7; 246.9]	24.5 [15.5; 58.0]	115.1 [52.5; 217.0]	**0.011**
CD8 TCM	42 [32.3; 56.3]	56.1 [23.6; 91.3]	70.5 [50.8; 131.4]	>0.05
CD8 Teff	126.1 [74.5; 156.1]	143.1 [39.1; 363.9]	67.2 [39.4; 232.9]	>0.05
CD8 TEM	162.7 [126; 176.1]	143.2 [110.7; 182.2]	183.2 [106.6; 359.3]	>0.05
DNT naïve	26.3 [15.8; 45.4]	15.0 [14.8; 24.9]	54.3 [31.1; 68.0]	>0.05
DNT TCM	4.5 [3; 6.3]	5.3 [2.4; 12.4]	7.4 [4.7; 9.6]	>0.05
DNT Teff	19.0 [5.8; 27.4]	11.3 [7.7; 19.6]	13.1 [3.3; 35.0]	>0.05
DNT TEM	5.7 [4.2; 12.3]	7.7 [3.2; 11.8]	5.6 [3.6; 9.4]	>0.05
CD19 DN	4.5 [2.5; 5.6]	5.4 [3.6; 8.7]	4.2 [2.6; 6.3]	>0.05
CD19 naïve	127.1 [89.1; 211.5]	113.0 [61.6; 145.9]	77.8 [43.2; 159.1]	>0.05
CD19 Sw	32.1 [22.7; 54.5]	41.7 [31.1; 51.7]	30.2 [22.8; 44.4]	>0.05
CD19 UnSw	20.3 [14.2; 22.8]	16.2 [10.7; 23.3]	17.5 [8.9; 29.9]	>0.05
**(B)**				
**Parameter,** **(10^3^/L)**	**UnV PN** **(*n* = 92)**	**AdV PN** **(*n* = 23)**	**InV PN** **(*n* = 24)**	***p*** **-value**
T0—acute phase				
CD4 naïve	236.62 [115.32; 432]	357.06 [151.2; 455.8]	246 [203.15; 368.1]	>0.05
CD4 TCM	213.68 [149.58; 372.5]	210.98 [157.1; 239.3]	296.6 [106.31; 483.2]	>0.05
CD4 Teff	14.79 [8.85; 24.8]	27.19 [8.24; 48.38]	14.1 [4.34; 39.6]	>0.05
CD4 TEM	173.25 [112.4; 222.6]	352.29 [217.9; 423.3]	204.7 [64.31; 322]	>0.05
CD8 naïve	51.28 [29.36; 81.6]	33.79 [26.45; 82.76]	68.6 [42.21; 134.4]	>0.05
CD8 TCM	24.81 [12.84; 57]	19.76 [10; 26.7]	54.6 [6.44; 89.4]	>0.05
CD8 Teff	93.23 [58.85; 169.5]	98.34 [72.26; 186.26]	62 [48.08; 72.6]	0.050
CD8 TEM	101.67 [55.97; 153.8]	126.01 [94.75; 150.06]	114 [52.56; 265.6]	>0.05
DNT naïve	8.52 [3.63; 15.9]	22.41 [11.48; 35.52]	18.8 [8.97; 40.7]	**0.024**
DNT TCM	2.05 [0.52; 5]	1.22 [0.56; 2.55]	2.4 [1.32; 15.2]	>0.05
DNT Teff	10.15 [5.8; 19.2]	16.23 [9.11; 22.02]	10.4 [5.36; 17.6]	>0.05
DNT TEM	3.41 [1.42; 5.6]	2.88 [1.01; 3.95]	3.3 [1.79; 8.9]	>0.05
CD19 DN	4.76 [3.07; 7.2]	5.73 [4.29; 9.75]	7.2 [4.58; 8.6]	>0.05
CD19 naïve	61.89 [47.92; 111.3]	77.22 [55.83; 161.63]	150.5 [37; 179.8]	>0.05
CD19 Sw	32.28 [20.9; 49]	30.75 [24.35; 48.24]	44.3 [32.26; 51.4]	>0.05
CD19 UnSw	10.69 [6.91; 27]	12.68 [8.47; 21.76]	12 [8.43; 29.8]	>0.05
1 month				
CD4 naïve	188.67 [123.18; 393.38]	425.3 [310.9; 636.3]	269.6 [222.9; 426.9]	>0.05
CD4 TCM	218.84 [177.18; 293.91]	367.28 [308.9; 486.5]	255.6 [201.7; 434.6]	**0.036**
CD4 Teff	18.97 [11.19; 27.37]	22.81 [7.57; 48.92]	12.43 [7.11; 34.91]	>0.05
CD4 TEM	285.61 [177.77; 352.14]	192.81 [145.1; 443.8]	217.3 [157.6; 313.9]	>0.05
CD8 naïve	38.55 [25.98; 73.53]	53.48 [25.52; 70.68]	76.03 [32.63; 123.9]	>0.05
CD8 TCM	39.7 [15.07; 57.35]	40.14 [25.64; 72.73]	45.75 [36.54; 69.6]	>0.05
CD8 Teff	129.58 [72.36; 253.26]	116.4 [77.04; 193.7]	117.9 [56.7; 272.8]	>0.05
CD8 TEM	176.84 [118.34; 252.63]	105.3 [91.8; 112.8]	161.4 [119.7; 250.1]	**0.022**
DNT naïve	16.02 [7.56; 36.66]	18.1 [16.06; 53.41]	16.6 [12.82; 24.59]	>0.05
DNT TCM	1.63 [0.87; 5.93]	5.38 [2.01; 8.06]	3.69 [1.55; 7.98]	>0.05
DNT Teff	15.45 [7.35; 36.4]	14.32 [11.15; 35.01]	12.91 [6.94; 25.92]	>0.05
DNT TEM	3.58 [1.64; 5.8]	2.97 [1.76; 7.44]	3.74 [2.75; 6.55]	>0.05
CD19 DN	5.48 [3.24; 6.76]	6.37 [3.29; 10.7]	4.44 [2.71; 6.33]	>0.05
CD19 naïve	67.81 [37.23; 106.12]	104.68 [67.74; 156.5]	69.47 [51.5; 99.14]	>0.05
CD19 Sw	27.61 [20.31; 41.14]	31.14 [25.76; 72.88]	28.08 [20.57; 36.36]	>0.05
CD19 UnSw	13.91 [7.39; 22]	33.61 [20.31; 38.92]	13.32 [8.86; 20.92]	**0.034**
6 months				
CD4 naïve	227.9 [139.3; 385.3]	239 [175.1; 386.6]	367 [196.3; 434]	>0.05
CD4 TCM	242.9 [165.7; 355.1]	221 [163.7; 306.4]	238.5 [194.4; 369]	>0.05
CD4 Teff	21.7 [10.9; 40.8]	22 [9.8; 35.2]	24.3 [13.6; 35.4]	>0.05
CD4 TEM	246 [187.5; 362.2]	206 [119.1; 351.7]	238.8 [189.7; 342]	>0.05
CD8 naïve	50 [26.2; 87.1]	65 [30.3; 136.8]	74.7 [49.3; 146.4]	**0.048**
CD8 TCM	42.6 [23.4; 75.1]	27 [16.7; 69.1]	52.6 [32.4; 79.6]	>0.05
CD8 Teff	140.7 [91.2; 222.8]	103 [67.3; 165.0]	141.5 [71.3; 289.1]	>0.05
CD8 TEM	187.7 [118.4; 264]	127 [62.5; 160.9]	184.6 [113.1; 231.8]	**0.036**
DNT naïve	18.1 [9.2; 34.9]	22 [7.7; 47.3]	20.8 [13.1; 31.2]	>0.05
DNT TCM	2.7 [1.4; 7.0]	3.1 [1.1; 7.0]	3.3 [2.4; 5.0]	>0.05
DNT Teff	17.1 [8.4; 35.7]	10.8 [8.1; 29.5]	12.9 [8.5; 22.9]	>0.05
DNT TEM	4.3 [2.5; 6.0]	3.3 [1.9; 8.2]	3.4 [2.5; 7.0]	>0.05
CD19 DN	4.55 [3.02; 7.01]	4.68 [3.57; 6.53]	3.38 [2.18; 5.91]	>0.05
CD19 naïve	85.74 [52.46; 160.84]	82.93 [67.96; 145.86]	82.88 [50.27; 118.23]	>0.05
CD19 Sw	31.56 [23.45; 42.92]	34.37 [19.82; 48.18]	42.41 [26.4; 50.81]	>0.05
CD19 UnSw	18.67 [13.29; 24.94]	19.46 [14.32; 33.41]	23.75 [11.47; 30.14]	>0.05
12 months				
CD4 naïve	255.16 [180.02; 433.18]	416.01 [326.92; 576.36]	356.88 [226.59; 438.28]	**0.020**
CD4 TCM	274.7 [193.09; 323.19]	287.59 [240.08; 346.69]	247.77 [201.16; 462.88]	>0.05
CD4 Teff	15.44 [7.9; 44.33]	20.13 [11.06; 51.5]	15.87 [9.49; 26.61]	>0.05
CD4 TEM	201.38 [146.12; 274.29]	246.02 [213.5; 391.16]	277.78 [213.92; 367.81]	**0.030**
CD8 naïve	63.68 [31.83; 95.17]	42.47 [35.11; 104.27]	80.97 [40.31; 118.01]	>0.05
CD8 TCM	47.17 [27.65; 76.03]	51.56 [32.24; 79.97]	48.19 [35.07; 77.27]	>0.05
CD8 Teff	164.83 [83.75; 225.94]	153.9 [93.63; 188.63]	104.69 [73.1; 217.17]	>0.05
CD8 TEM	159.77 [97.83; 269.96]	137.3 [112.84; 246.39]	166 [108.1; 252.08]	>0.05
DNT naïve	20.88 [10.94; 38.07]	29.14 [17.86; 66.47]	20.34 [14.78; 36.5]	>0.05
DNT TCM	3.66 [1.63; 5.6]	5.92 [2.56; 16.54]	3.82 [2.33; 5.84]	>0.05
DNT Teff	14.37 [7.43; 26.24]	14.77 [13.04; 32.65]	10.13 [5.86; 19.34]	>0.05
DNT TEM	4.44 [2.28; 6.93]	4.01 [2.28; 8.63]	5.17 [3.75; 6.4]	>0.05
CD19 DN	5.19 [3.72; 7.2]	5.72 [4.41; 9.3]	4.89 [2.93; 6.23]	>0.05
CD19 naïve	95 [58.35; 142.45]	117.23 [87.64; 193.5]	135.1 [119.95; 190.13]	>0.05
CD19 Sw	36.43 [26.73; 46.07]	45.78 [28.25; 77.56]	35.93 [27.74; 54.21]	>0.05
CD19 UnSw	20.39 [16.07; 29.9]	24.83 [14.22; 37.52]	20.84 [13.57; 23.92]	>0.05

**Notes:** (A) Participants without pneumonia (NonPN); (B) participants with pneumonia (PN). Data are presented as median [Q1; Q3]. T0 represents the acute phase; subsequent assessments were performed at 1, 6, and 12 months. Absolute counts of T- and B-cell subsets are expressed as 10^3^/L. Bold values indicate statistically significant differences among the three groups (*p* < 0.05). **Abbreviations:** AdV, adenovirus-vectored vaccine; CD, cluster of differentiation; DN, double-negative; DNT, double-negative T cells; InV, inactivated vaccine; NonPN, without pneumonia; PN, pneumonia; Sw, switched memory B cells; TCM, central memory T cells; Teff, effector T cells; TEM, effector memory T cells; UnSw, unswitched memory B cells; UnV, unvaccinated.

**Table 4:** Linear mixed-effects modeling of longitudinal naïve CD8+ T-cell dynamics across vaccine groups.

Fixed Effect	F	*p*-Value
**AdV**		
Time Point	4.082	0.013
Pneumonia	0.007	0.933
Age	40.143	**0.0001**
TP × Age	3.812	0.017
**InV**		
Time Point	1.034	0.387
Pneumonia	0.179	0.675
Age	25.925	**0.0001**
TP × Age	0.642	0.592
**UnV**		
Time Point	1.924	0.130
Pneumonia	8.651	**0.004**
Age	72.405	**0.0001**
TP × Age	1.036	0.380

**Notes:** Linear mixed-effects models were used to assess longitudinal changes in naïve CD8^+^ T-cell counts across time points within each vaccine group. Fixed effects included time point, pneumonia status, age, and the time point × age interaction. Bold values indicate statistical significance (*p* < 0.05). **Abbreviations:** AdV, adenovirus-vectored vaccine; InV, inactivated vaccine; UnV, unvaccinated; TP, time point; *F*, F-statistic.

**Table 5:** Characteristics of the quality of models.

Model	Class	Precision	Recall	F1-Score	Support (n)
Vaccination Group Classification	InV (*n* = 35)	0.86	0.33	0.48	54
	UnV (*n* = 112)	0.65	0.98	0.78	132
	AdV (*n* = 38)	0.92	0.24	0.38	46
	Overall Accuracy				68%
	Macro Average	0.81	0.52	0.55	232
	Weighted Average	0.75	0.68	0.63	232
Vaccination + Pneumonia Classification	InV_NonPN (*n* = 11)	1.0	0.46	0.63	13
	InV_PN (*n* = 24)	0.55	0.26	0.35	23
	UnV_NonPN (*n* = 20)	0.64	0.35	0.45	20
	UnV_PN (*n* = 92)	0.57	0.96	0.71	68
	AdV_NonPN (*n* = 15)	0.71	0.29	0.42	17
	AdV_PN (*n* = 23)	1.0	0.36	0.53	14
	Overall Accuracy				61%
	Macro Average	0.74	0.45	0.51	155
	Weighted Average	0.66	0.61	0.57	155

**Notes:** Precision, recall, and F1-score are reported for each class. Support indicates the number of observations in the test set for each class. Overall accuracy represents the proportion of correctly classified observations. Macro average represents the unweighted mean across classes, whereas weighted average accounts for class-specific support. *n* indicates the total number of participants in each study group. **Abbreviations:** AdV, adenovirus-vectored vaccine; InV, inactivated vaccine; NonPN, without pneumonia; PN, pneumonia; UnV, unvaccinated.

## Data Availability

The data presented in this study are available on request from the corresponding author because the project was funded by the government and remains subject to financial audits and data verification procedures even five years after its completion.

## Supplementary Materials

The following supporting information can be downloaded at https://www.mdpi.com/article/10.3390/microorganisms14102252/s1, Figure S1: Levels of binding and RBD-specific (neutralizing) IgG over 12 months in the study groups; Figure S2: Dynamics of the CD4^+^ memory-cells population over 12 months; Figure S3: Dynamics of the CD8^+^ memory-cells population over 12 months; Figure S4: Dynamics of the DNT memory-cells population over 12 months; Figure S5: Dynamics of the CD19^+^ memory-cells population over 12 months.

## Abbreviations

The following abbreviations are used in this manuscript:

AdV	Adenovirus-vectored vaccine
AI	Adenovirus-vectored vaccine vs. inactivated vaccine
AU	Adenovirus-vectored vaccine vs. unvaccinated group
AUC	Area under the curve
CD	Cluster of differentiation
CI	Confidence interval
CRP	C-reactive protein
DN	Double-negative
DNT	Double-negative T cell
FDR	False discovery rate
IgG	Immunoglobulin G
IgM	Immunoglobulin M
InV	Inactivated vaccine
LME	Linear mixed-effects
NonPN	Without-pneumonia group
PBMC	Peripheral blood mononuclear cell
PCR	Polymerase chain reaction
PN	Pneumonia group
RBD	Receptor-binding domain
REML	Restricted maximum likelihood
ROC	Receiver operating characteristic
SARS-CoV-2	Severe acute respiratory syndrome coronavirus 2
SHAP	SHapley Additive exPlanations
Sw	Switched memory
TCM	Central memory T cell
Teff	Effector T cell
TEM	Effector memory T cell
UI	Unvaccinated group vs. inactivated vaccine
UnSw	Unswitched memory
UnV	Unvaccinated group
WBC	White blood cell count
